# Evaluation of Strategies to Improve the Environmental and Economic Sustainability of Cow–Calf Production Systems

**DOI:** 10.3390/ani12030385

**Published:** 2022-02-05

**Authors:** Phillip A. Lancaster, Robert L. Larson

**Affiliations:** Beef Cattle Institute, Kansas State University, Manhattan, KS 66506, USA; rlarson@vet.k-state.edu

**Keywords:** beef cattle, economics, environment, sustainability, systems modeling

## Abstract

**Simple Summary:**

Beef cattle have a significant contribution to greenhouse gas emissions globally, but they have a unique ability to digest plant material that is inedible for humans, thus producing human food from grasslands and rangelands. Additionally, many people around the world depend upon cattle ranching of grasslands and rangelands for their livelihoods. Identifying the strategies likely to have the largest impact on greenhouse gas emissions while improving or maintaining economic returns is necessary to guide future research. The goal of the current study was to evaluate four potential strategies for improving the environmental and economic sustainability of cow–calf production. The four strategies included (1) decreasing the feed required for maintenance, thus increasing the feed available for growth, (2) decreasing the time for cows to rebreed after calving, (3) increasing the digestibility of pasture grass, and (4) increasing the yield of pasture grass. A computer simulation model of a cow herd in Kansas, U.S.A., was modified to create variation in the four strategies. Decreasing the feed required for maintenance improved both environmental and economic sustainability, and increasing the yield of pasture grass improved economic sustainability, implying that these strategies should be primary targets to enhance the sustainability of cow–calf production systems.

**Abstract:**

Grazing cow–calf production systems account for 60 to 70% of the greenhouse gas emissions of U.S. beef production. The objective of this analysis was to evaluate the importance of management strategies (cow maintenance energy requirements, reproductive efficiency, forage nutritive value, and forage yield) on the sustainability of cow–calf production systems using a sensitivity analysis in a production systems model. The Beef Cattle Systems Model was used to simulate a cow–calf production system in the Kansas Flint Hills using Angus genetics over a 24 year time period. The model was modified to create variation among cow herds in the base net energy for the maintenance requirement (NEm_Req), postpartum interval (PPI), grazed forage digestibility (Forage_TDN), and forage yield per hectare (Forage_Yield). The model was run for 1000 iterations/herds of a 100-cow herd. A stepwise regression analysis in conjunction with standardized regression analysis was used to identify important predictors of an indicator of greenhouse gas (GHG) emission intensity, dry matter intake per kilogram weaned, and two indicators of economic sustainability, winter feed use and returns over variable costs, using R statistical software. The most important predictor of DMI per kilogram weaned was calf weaning weight followed by NEm_Req, whereas returns over variable costs were primarily influenced by kilograms weaned per cow exposed and total purchased feed (supplement + winter feed), which were strongly influenced by NEm_Req and Forage_Yield, respectively. In conclusion, decreasing the net energy required for maintenance improved both economic and environmental sustainability, and increasing forage yield and length of the grazing season improved economic sustainability, implying that these strategies should be primary targets to enhance the sustainability of cow–calf production systems.

## 1. Introduction

Livestock production accounts for 14.5% of anthropogenic greenhouse gas (GHG) emissions globally, with beef production contributing 41% of the total livestock emissions [1]. Beef production uses more natural resources (land, feed, water) than other livestock production systems, but 40% of land area globally is in grassland or rangelands, which can be used for feed production for cattle [2,3]. However, the lesser efficiency of natural resource use in beef production systems compared with swine and poultry production systems requires the development of more sustainable beef production systems. For example, production systems that utilize concentrate feeds for post-weaning growing diets in addition to forage for reproducing cows and pre-weaning calves reduce the GHG emission intensity of beef production [4,5,6,7], and countries where concentrate feeding of post-weaning growing cattle is common have lesser GHG emission intensities of beef production [8].

In the current U.S. production system, the cow–calf sector of the industry accounts for 60 to 70% of the GHG emission intensity for beef production [9,10], indicating that improvements in sustainability of this sector are important for improving the environmental sustainability of beef production. Methane emissions from the fermentation of low-quality forage is the major contributor to the GHG emission intensity of the cow–calf sector, and 70% of feed consumed by brood cows is used for maintenance energy requirements [11]. Reducing maintenance energy requirements would improve conversion of feed to beef, thus decreasing GHG emission intensity [6,12]. Additionally, reproductive traits are reported to have 4 to 10 times greater importance to the economic sustainability of beef production than growth and carcass traits [13,14,15], but grazing management and forage yield impact winter feed costs, which are a major driver of profitability [16,17,18,19]. Previous research has evaluated differences in cow size and maintenance energy requirements [12,20,21], reproductive efficiency [21,22], and grazing management [23,24,25] on the efficiency and GHG emission intensity of beef production, but the impact of these parameters on environmental and economic sustainability has not been evaluated simultaneously.

Systems models are important tools for evaluating the impacts of management decisions on outcomes of complex systems [26] and have been used to model beef production systems extensively [27,28,29,30,31,32,33,34,35]. Experimental methods to evaluate the multifaceted components that could increase the environmental and economic sustainability of beef production would be prohibitively expensive and time consuming. The objective of this analysis was to evaluate the importance of cow maintenance energy requirements, reproductive efficiency, forage nutritive value, and forage yield on the sustainability of cow–calf production systems using a sensitivity analysis in a cow–calf production systems model.

## 2. Materials and Methods

### 2.1. Beef Cattle Systems Model (BCSM)

The model represents a cow–calf production system modeled at the individual animal level on a daily time-step. Animal characteristics determined daily include age, weight, body condition score (BCS), lactation, nutrition requirements, nutrient availability, reproductive status, morbidity, and mortality. The daily outcomes of these traits are determined by interactions among the animal’s genetics, the previous day’s trait outcomes, variable stochasticity, the animal’s production phase and state, calendar date, and the application of model parameters. Model parameters can be found in the supplementary tables. Linear production phases for animals that stay in the herd include (in order by age) nursing calf, post-weaning non-pregnant replacement heifer, bred replacement heifer, two-year-old cow, three-year-old cow, and mature cow (≥four years old). Recurring production states include lactating and non-lactating, cycling and non-cycling, and pregnant and non-pregnant. Simulated management decisions such as culling, replacement, feed source, and exposure for breeding are made carried out daily contingent on the calendar date and feedback provided by animal characteristics at the individual and aggregate herd level.

When designing BCSM, the developers [36] considered two mutually exclusive options to account for the interaction of feed expenses, BCS, and postpartum interval (PPI; and the effect of PPI on reproductive efficiency) as drivers of herd efficiency when measured by kg weaned per cow exposed divided by feed costs. One option would be to hold feed expenses static, allowing changes in BCS to impact PPI and subsequently reproductive efficiency, resulting in holding the denominator constant while allowing the numerator (kg weaned per cow exposed) to vary. The alternative option would be to hold BCS static by providing necessary nutrients regardless of the cost, resulting in minimal variation in PPI and reproductive efficiency, resulting in the numerator being held relatively constant while allowing the denominator (feed cost) to vary. We chose the latter option; therefore, when interpreting the output of the model, feed costs are allowed to vary greatly, while BCS, PPI, and reproductive efficiency will have limited variability.

A full description of the BCSM functionality is provided by Aherin [36]. Briefly, the model simulates a herd of 100 breeding females exposed to bulls for 63 days each year as heifer and cow breeding season starts, and end dates are identical. A “production year”, as defined in the model, is the time from calving in year *i* to either calving in year *i* + 1 (calving interval) or culling for each individual breeding female; thus, the length of each production year will vary among individual animals. Sixty days after the end of the breeding season, pregnancy status is determined, and all non-pregnant females are sold at calf weaning. Additional culling occurs based on minimum levels set for each cow age group. If the percent of non-pregnant females within an age group has not reached the minimum level, voluntary culling (assumed to result from disposition, foot quality, udder quality, etc.) occurs until the minimum for each specific age group is reached. All cows 13 years old at the time of pregnancy detection are culled on the cull date. Any female that aborts a pregnancy after the cull date is culled on that day. If a calf dies between calving and the start of the breeding season, the breeding female is removed from the herd at this time or at any day corresponding with the female’s own mortality. Heifer calves are kept to replace the culled females plus an additional calculated number from past cow losses (mortality, abortion, calf-loss before breeding season) that occur between weaning and the next breeding season. Replacement heifers are selected in the order from oldest to youngest. If the number of raised heifer calves is insufficient to meet replacement heifer requirements, non-pregnant replacement heifers are purchased with traits matching the raised heifer population. All calves are weaned on the same date and sold on the weaning date. The weaning date is set as the date upon which the oldest calf is 220 days old.

### 2.2. BCSM Parameterization for Current Analysis

Four parameters representing the 4 strategies were varied to evaluate the effects of cow maintenance energy requirements, reproductive efficiency, forage nutritive value, and forage yield per hectare on key outcomes impacting sustainability. In the model, a distribution was created using a mean and standard deviation of each parameter from which the model drew a single value for each herd (iteration). These parameters were different for each herd (iteration) but constant throughout the 24-year simulation of a given herd (iteration). Variation in the base net energy for maintenance of an individual animal was modeled using a random normal distribution with mean of 0.077 and standard deviation of 0.002 Mcal/kg^75^ of shrunk body weight. The BCSM randomly selected a value (NEm_Req) from this distribution for each herd, where the same value was applied to every animal in the herd (cows, replacement heifers, calves), which was used to compute net energy for maintenance requirements, feed required for maintenance, and feed available for gain. In the BCSM, postpartum interval of individual cows within a herd is drawn from a distribution based on body condition score at calving (Table S9). Variation in reproductive efficiency for a herd/iteration was modeled by adjusting the postpartum interval distribution in the BCSM. A random normal distribution with a mean of 0 and standard deviation of 4 days was used. The BCSM randomly selected a value (PPI_Addend) as an integer from this distribution for each herd, which was added to the minimum, maximum, and mode of the original distribution used to select the postpartum interval of reproductive females. Each female in the herd received a PPI based on BCS at calving in each year. A random normal distribution with mean of 1 and standard deviation of 0.02 was used to model variation in forage TDN. The BCSM randomly selected a value (Forage_TDN) from this distribution for each herd, which was multiplied by each monthly forage TDN value in the input file. A random normal distribution with mean of 1 and standard deviation of 0.033 was used to model variation in forage yield. The BCSM randomly selected a value (Forage_Yield) from this distribution for each herd, which was multiplied by each annual predicted forage yield.

The BCSM was parameterized for spring-calving Angus cows grazing Kansas Flint Hills native prairie from 1995 to 2018 using EPDs from American Angus Association, available data on forage yield per acre and forage digestibility, and historical Kansas Mesonet weather data for Manhattan, Kansas, USA. Financial parameters were monthly feeder calf prices, average 85–90% lean cull cow prices, and feed prices from the Livestock Marketing Information Center [37], pasture rent from Kansas Bluestem Pasture Survey [38], and monthly agriculture interest rates from the Kansas City Federal Reserve [39]. Cattle were allowed to graze each year starting May 1 and ending when residual forage reached 50% of forage yield, assuming a conservative stocking rate that was expected to result in 25% of forage yield consumed, 25% trampled, and 50% residual. When forage digestibility declined below 50%, a protein supplement (45% CP; 28% RDP) was supplied at 0.1% of shrunk body weight and forage digestibility was adjusted based on previous research [40,41,42,43,44,45,46,47]. A winter feed ration with TDN of 57% was used when residual forage reached 50% of forage yield. An energy supplement with TDN of 70% was used to meet energy deficits for maintenance or gain when forage or winter feed ration alone did not meet net energy requirements.

### 2.3. Sensitivity Analysis

The BCSM was run for 1000 iterations/herds for a 100-cow herd. The initial 9 years were removed, and data analysis was performed on the remaining 15 years. Outputs were averaged across years for each iteration because the variables of interest (NEm_Req, PPI, Forage_TDN, Forage_Yield) were implemented on an iteration/herd level. Descriptive statistics of output variables were computed using *describe* function of R statistical software (version 4.0.4; R Core Team, 2021), and standardized regression coefficients computed using *lm.beta* function for regression of model outputs on NEm_Req, PPI, Forage_TDN, and Forage_Yield. Additionally, regression analysis was used to determine importance of production parameters on indicators of GHG emission intensity and profitability. Enteric and manure methane (58 to 68%) and manure nitrous oxide (22 to 25%) emissions are associated with feed digestion and account for 80 to 93% of total GHG emissions of the cow–calf sector [9,10], indicating that feed intake is a reasonable proxy of GHG emissions. Thus, dry matter intake per kilogram weaned was used as an indicator of GHG emission intensity. Non-pasture feed costs, the majority of which goes towards winter feed, account for 40% of total variable costs, and the most profitable one-third of cow–calf operations had 50% lower non-pasture feed costs compared to the least profitable one-third according to data from Kansas Farm Management Association (KFMA) [48]. Thus, winter feed use and returns over variable costs were used as indicators of profitability. Stepwise regression procedures in conjunction with standardized regression analysis was used to determine the importance of production parameters on indicators of GHG emission intensity and profitability. First, Pearson correlation coefficients were computed using *corr* function of R between variables of interest and model outputs. Model outputs with correlation coefficients greater than 0.30 were included in stepwise regression using *stepAIC* function to select the model with the lowest Akaike information criterion (AIC) value, then variance inflation factors were computed using *vif* function. If any predictors had variance inflation factor (VIF) values greater than 10, then a sequential procedure was used to remove the variable with the greatest VIF value, the regression model was rerun, and VIF values were reevaluated until all predictors had VIF values less than 10. Standardized regression coefficients were computed for the final model as described above. Standardized regression coefficients less than 0.30 were considered weak, 0.31 to 0.50 moderate, and ≥0.51 indicated a strong influence on the output variable.

## 3. Results and Discussions

Profitability and return on investment, as well as environmental impact, are important aspects of cattle ranching that require improvement for cow–calf production to be sustainable. Many facets of cow–calf production impact environmental and economic sustainability, but identifying those that have the largest impact and should receive heightened focus is needed for rapid improvements. This analysis was undertaken to determine the relative importance of production parameters that could be changed to enhance environmental and economic sustainability.

The base NEm requirement was varied to simulate making genetic changes in efficiency of energy use because forage and feed digestion is the largest driver of GHG emissions [9,10] and total variable costs [49,50]. In our model, animals consumed the same amount of forage or feed based on body weight regardless of NEm_Req, and thus, the amount of feed available for gain (i.e., NEΔ or NEg) varied with changing NEm requirement. The mean (SD) base NEm requirement was 0.0769 (0.0019) Mcal/kg BW^75^, which is similar to the base NEm requirement for Bos taurus cattle in the National Academy of Science, Engineering, and Medicine (NASEM) [51] by design (Table 1).

The SD represents a coefficient of variation of 2.5%, but there are few data available on the variation in fasting heat production. The NASEM [51] uses a base NEm requirement that is 10% less for Bos indicus breeds and 20% greater for high-milking breeds, and Hotovy et al. [52] reported that genetic variation exists in fasting heat production with a range among individuals of approximately 0.073 to 0.088 Mcal/kg BW^75^. Thus, the distribution used in this simulation is similar to that reported by Hotovy et al. [52], giving values within ±8%.

Forage TDN was varied to simulate making changes in forage digestibility either through plant breeding or the development of new feed additives. The Forage_TDN multiplicative factor had an SD of 0.021 with a range of 0.937 to 1.088. The NASEM [51] reported a coefficient of variation for the TDN of native prairie hay of 9.8% based on 130 samples, and Olson et al. [53] reported a coefficient of variation in gross energy digestibility of 10.0% for 13 native prairie hay samples. The distribution chosen for Forage_TDN is within the range of previously reported values of energy digestibility.

The postpartum interval was varied to simulate making changes in reproductive efficiency either through genetics or management. The PPI_Addend had an SD of 3.57 days with a range of −12 to +11. The mean PPI ranged from 55.5 to 66.7 days among sire breeds for cows in good body condition [54], and Crowe et al. [55] reported that the PPI can range from 20 to 120 days in individual beef cows. In the current simulation, the PPI could range from 33 to 86 days for individual cows with BCS 5, and the mean PPI for herds with BCS 5 could range from 48 to 71 d, which is within the range of reported values.

Forage yield varies across years due to weather, which is captured in the forage yield prediction equation used in the BCSM, but the equation produces the same annual forage yield in each year for each herd simulated. Forage yield was varied to simulate increasing stocking rate and grazing days, as well as reducing land use for purchased feed production. The Forage_Yield multiplicative factor had an SD of 0.035 with a range of 0.911 to 1.096. Owensby et al. [56] and Teague et al. [24] reported an 8 to 33% increase in forage yield, but Briske et al. [57] and Augustine et al. [58] reported no increase in forage yield with management of intensive grazing in tallgrass native prairie. Thus, the range of ±10% used in this simulation is at the lower end of reported values.

Descriptive statistics for BCSM outputs of interest are presented in Table 2. The mean percentage of cows cycling in first 21 days of the breeding season, pregnancy percentage, and percentage of cows calved in first 21 days of the calving season were 88.1, 93.0, and 59.4%, respectively, which agree with experimental results [59,60,61,62]. At calving, the BCS of cows ranged from 5 to 6, while at weaning, the BCS of cows ranged from 4 to 5, and weaned calves had a mean weight of 207.8 kg. Cows required an average of 421 kg of supplemental feed throughout the year and 1850 kg of winter feed ration during the 6 months without grazing. These BCSM outputs are in line with standard performance analysis data [48,63,64].

Several measures were evaluated in the BCSM to evaluate the efficiency of production and the environmental impact, which is highly correlated with efficiency [65,66]. Dry matter intake per kilogram weaned averaged 26.49 kg/kg and is an indicator of GHG emission intensity as methane and nitrous oxide from forage and feed digestion account for 80 to 93% of the carbon equivalent emissions of the cow–calf sector [9,10]. The amount of GHG emitted from forage and feed digestion are likely to be different among production systems, but within a given production system the amount of forage and feed used will be strongly, positively related to the GHG emissions. Kilogram weaned per cow exposed averaged 172.4 kg and is an important measure of production efficiency, which is slightly lesser than that reported in standard performance analyses [48] (190 to 227 kg) [63,64]. The reason for the lower weaning weights estimated by the BCSM is unclear but is likely due to underestimation of forage intake or the efficiency of gain in young calves by the available prediction equations [67]. Kilogram weaned per grazed hectare averaged 60.7 kg/ha and is a measure of efficiency of grazing land use, which agrees with performance analysis data from the Kansas Farm Management Association (KFMA) [48].

On average, gross revenue and total variable costs were USD 708.12 and 667.82 per cow, resulting in returns over variable costs of USD 40.30 per cow and USD 14.27 per hectare, and a return on investment of 8%. Winter feed cost was USD 307.81 per cow and accounted for 46% of total variable costs. The KFMA reported similar values with a revenue of USD 773.04, feed cost of USD 290.07, and total variable cost of USD 749.26 per cow from 2015 to 2019.

The PPI of the herd strongly influenced reproductive measures of percentage cycling in the first 21 days of the breeding season, pregnancy percentage, percentage calved in first 21 days of the calving season, mean cull cow age, and percentage of replacement heifers required with standardized regression coefficients of −0.96, −0.59, −0.77, −0.51, and 0.46, respectively (Table 3). This result was expected given that the probability of a cow becoming pregnant in a defined breeding season is highly dependent upon her return to estrus after calving. In contrast, NEm_Req, Forage_TDN, and Forage_Yield had little influence on reproductive measures with standardized regression coefficients ranging from −0.05 to 0.08. It was expected that reduced maintenance energy requirements or increased forage TDN would allow the cow to maintain body condition through weaning and the next calving; however, the BCSM was configured so that individual cows are maintained at no less than BCS 4 during lactation and fed to achieve BCS 5 to 6 at calving, which diminished the effects of nutrition on reproduction.

Calf weaning weight and the supplement and winter feed fed to cows were more highly influenced by NEm_Req, Forage_TDN, and Forage_Yield than PPI. For actual weaning weight, NEm_Req had the greatest influence (−0.66) followed closely by Forage_TDN (0.58) such that reducing NEm_Req or increasing Forage_TDN results in increased weaning weights of calves. Likewise, NEm_Req (0.71) and Forage_TDN (−0.58) had a greater influence on the supplemental feed fed to cows than Forage_Yield (0.22). Increasing NEm_Req and decreasing Forage_TDN resulted in greater amounts of supplemental feed fed to cows, which is expected, but increasing Forage_Yield also resulted in greater amounts of supplemental feed fed to cows. The reason for this has to do with the length of the grazing season being greater with increasing Forage_Yield, because forage digestibility declines as warm-season native prairie species mature and enter dormancy later in the grazing season, and more supplemental feed will be required to maintain body condition. Conversely, the amount of winter feed fed to cows was more influenced by Forage_Yield (−0.65) than NEm_Req (−0.47) or Forage_TDN (0.44). Increasing forage yield per hectare reduced winter feed fed because the length of the winter feeding period was reduced, but the relationship of winter feed with NEm_Req and Forage_TDN was initially surprising. However, based on the NASEM [51] feed intake equation, DMI increases as the NEm density, and likewise the TDN, of the diet increases; thus, as Forage_TDN increased the DMI of cows increased, resulting in shorter grazing seasons and longer winter feeding periods, which can be seen in the negative relationship with grazing days per hectare. Decreasing NEm_Req resulted in greater amounts of winter feed fed for a couple of reasons. Cows and calves with lesser maintenance requirements have a greater proportion of DMI available for gain, resulting in heavier animals that consume more forage, thus shortening the grazing season, which can be seen in the positive relationship between NEm_Req and grazing days per hectare and negative relationships between NEm_Req and calf weaning weight (−0.66) and BCS at weaning of 2-year-old (−0.45), 3-year-old (−0.32), and mature (−0.48) cows. Furthermore, cows with less maintenance requirements also gain more weight while consuming winter feed, resulting in heavier animals that consume more winter feed, which can be seen in the negative relationships between NEm_Req and BCS at calving of 2-yr-old (−0.16), 3-yr-old (−0.23), and mature (−0.67) cows.

The relationships of NEm_Req with the amount of supplement and winter feed are unexpected and are a result of the BCSM predicting the DMI of animals based on shrunk body weight regardless of the base NEm requirement. The NASEM [51] uses adjustment factors to decrease or increase the net energy for maintenance of indicine and high-milking cattle breeds, respectively, compared with taurine breeds. However, the NASEM [51] does not adjust the DMI equation for Indicine or high-milking breeds relative to Taurine breeds. Huffman et al. [68] and Elzo et al. [69] reported greater feedlot DMI with increasing Brahman percentage in Brahman × Angus steers, but Elzo et al. [69] reported a lower DMI for 100% Brahman steers. Ferrell and Jenkins [70] reported no difference in DMI per steer per day between Angus and Brahman-sired calves, and no difference in DMI as percentage of BW among all breeds. Abney [71] reported that yearling Holstein steers had a greater DMI per day than yearling Angus steers, but not DMI as a percentage of BW. In contrast, calf-fed Holstein steers has a lower DMI per day and as percentage of BW than calf-fed Angus steers. Thus, there does not appear to be a clear relationship between net energy for maintenance requirement and feed intake that could be used to adjust the DMI prediction equation. Because of the lack of an established relationship between net energy for maintenance requirement and feed intake, no adjustment to the DMI equation in the BCSM was carried out to reflect changes in NEm_Req.

Several measures of efficiency were evaluated, and the importance of NEm_Req, Forage_TDN, PPI, and Forage_Yield within the modeled parameter distributions was determined. Forage yield had the greatest influence on grazing days per hectare such that increasing forage yield resulted in a greater number of grazing days per hectare, as would be expected. Interestingly, Forage_TDN had the second-greatest influence on grazing days per hectare such that greater forage TDN resulted in fewer grazing days per hectare. The reason for this is that the NASEM [51] feed intake equation predicts a greater DMI with increasing dietary net energy concentration, resulting in greater DMI per cow per day. Kilogram weaned per cow exposed and kilogram weaned per hectare were influenced, in order from the greatest impact to the least, by NEm_Req, Forage_TDN, and PPI such that decreasing NEm_Req or PPI and increasing Forage_TDN increased kilogram weaned per cow exposed or per hectare. Dry matter intake per kilogram weaned was strongly influenced by NEm_Req and Forage_TDN (Table 3). Decreasing NEm_Req and increasing Forage_TDN decreased DMI per kilogram weaned by increasing the net energy available for gain and improving efficiency. Postpartum interval and Forage_Yield had less influence on DMI per kilogram weaned.

The net energy for maintenance requirement and Forage_TDN had the greatest influence on total revenue per cow as both decreasing NEm_Req and increasing Forage_TDN result in a greater net energy for gain intake and calf weaning weight. Forage yield and NEm_Req had the greatest influence on fed ration cost, interest expense, and total variable cost, although the influence was only weak to moderate. Increasing Forage_Yield or decreasing NEm_Req decreased these expenses. Replacement cost was most influenced by PPI followed by a weak influence of NEm_Req and Forage_TDN. Returns over variable costs per cow or per hectare and annual return on investment were moderately influenced by NEm_Req and weakly influenced by Forage_TDN and Forage_Yield; PPI had minimal influence. Decreasing NEm_Req and increasing Forage_TDN or Forage_Yield increased all three measures of profitability.

Dry matter intake per kilogram weaned is an important measure of efficiency as it is directly and highly related to GHG intensity as discussed previously; thus, further analysis was performed to better understand the factors influencing this variable. Regression analysis revealed that the final stepwise model explained 97.7% of the variation in DMI per kilogram weaned (Table 4). Standardized regression analysis of these variables indicated that calf weaning weight has the greatest influence followed by NEm_Req. Several variables, including Forage_TDN, the percentage of replacement heifers required, total net energy for gain intake, and calf mortality, had a weak influence on DMI per kilogram weaned, while Forage_Yield, pregnancy percentage, and DMI per day per cow had minimal influence on DMI per kilogram weaned. Similar to our results, Baber et al. [12] reported that using a terminal crossbreeding system that increased weaning weights by 10% reduced greenhouse gas emission intensity by 6.5% and reduced mature cow size by 20% while weaning weights remained constant (i.e., similar to reducing the net energy for maintenance requirements of the cow herd), and greenhouse gas emission intensity decreased by 6%. In contrast to the current results, increasing fiber digestibility, which would be similar to changing Forage_TDN, had minimal impact on GHG emission intensity [12].

Winter feed cost is a major expense in cow–calf production systems due to the lack of quantity and poor nutritive value of dormant forage and accounted for 46% of the total variable expense in the current analysis. Performance analysis [48,63,64] indicates that the most profitable herds have the lowest costs. Feed price is a major component of feed cost, but management changes also influence the amount of winter feed used. Thus, further analysis of factors influencing winter feed use was performed. Regression analysis revealed several variables that explained 98.4% of the variation in winter feed use. Of these variables, grazing days per hectare had a strong impact on winter feed use with greater grazing days reducing winter feed use. The net energy for maintenance had a weak influence with less NEm_Req resulting in greater winter feed use for the reasons described above. Forage yield and kilogram weaned per cow exposed had a weak influence and a positive relationship with winter feed use. The percentage of replacement heifers required, percentage of cows calving in the first 21 d, PPI, and pregnancy percentage had minimal influence on winter feed use.

Returns over variable costs are a direct measure of profitability and important measure of economic sustainability. Regression analysis revealed several predictors that explained 37.7% of the variation in returns over variable costs. The reason for the lower coefficient of determination for returns compared with DMI per kilogram weaned and winter feed use is that feed prices were excluded from the analysis of returns, which likely has a large influence on the variation. Kilogram weaned per cow exposed and total purchased feed (supplement + winter feed) had a moderate influence on returns over variable costs where increasing kilogram weaned per cow exposed and decreasing total purchased feed increased returns. When analyzing these variables, decreasing NEm_Req and increasing Forage_TDN had a strong influence on increasing kilogram weaned per cow exposed, whereas increasing Forage_Yield had a strong influence (−0.86), and decreasing NEm_Req had a weak influence (0.31) on total purchased feed. The percentage of replacement heifers required and pounds of cull cows sold had a weak influence, and the percentage of cows calving in the first 21 d, grazing hectares per cow, and PPI had minimal influence on returns over variable costs.

Postpartum interval had a strong influence on all of the reproductive outputs, but PPI and the reproductive outputs had a weak to minimal influence on DMI per kilogram weaned and returns over variable costs, indicating minimal impact on environmental and economic sustainability under the constraints of this model that prevented PPI from reaching long, but possible lengths and subsequent low reproductive outputs. The primary reason is the BCSM was designed to achieve optimal reproductive performance. The PPI ranged from 45 to 70 d, and almost 90% of cows were cycling in the first 21 days of the breeding season, resulting in a coefficient of variation for pregnancy percentage of 1% and range of 89 to 96%. This lack of variation constrains the ability of reproductive outcomes to influence other parameters and indicates that while PPI was longer than that modeled, it may have an important impact on efficiency and sustainability, and improvements over current good management practices may yield negligible benefits. Globally, feed availability can be limiting in some production environments such that feeding cows for optimal reproductive performance is not feasible, and thus, the results of this analysis may be more applicable to production environments where feed availability is not limiting. However, the impact of the four strategies to improve sustainability would have similar relevance to the variation in reproductive efficiency if feed availability was limited in the model, because in the model, nutrition is directly tied to BCS, which directly impacts PPI.

Forage is the primary and typically the least expensive feed resource available to the cow–calf enterprise, and improving the effectiveness of this resource could have significant impacts on sustainability. One of the metrics evaluated, Forage_Yield, was very influential on changing grazing days per hectare and subsequently on winter feed use and returns over variable costs, resulting in improvements in economic sustainability. Thus, strategies to increase forage production through plant genetics or to increase forage the harvest efficiency of the animal through grazing management would have a significant impact on reducing winter feed use and increasing returns over variable costs. However, Forage_Yield had a minimal influence on DMI per kilogram weaned, a measure of environmental sustainability.

Improving forage digestibility through plant breeding, animal breeding, or the use of feed additives is another approach to enhance the utilization of the forage resource. Increasing forage digestibility had a weak influence on increasing returns over variable costs, probably through increased calf growth and revenue, even though it had a moderate influence on increasing winter feed costs. Thus, there may be an optimum forage digestibility in a cow–calf production system to achieve maximum economic sustainability because excessive forage digestibility could result in greater forage intake by cows and subsequently shorter grazing seasons and increased winter feed costs. Increased forage digestibility decreased DMI per kilogram weaned, probably through the strong influence on calf weaning weight, and thus likely reduces the greenhouse gas emission intensity of the cow–calf production system. Another alternative could be plant breeding strategies to improve the digestibility of more mature forages as the largest deficit in grazed forage energy consumption for the both the cow and the calf occurs later in the grazing season, which coincides with increased plant maturity. Forage cell wall concentration increases as the plant matures and has a large influence on dry matter digestibility [72], and lignin concentration, which also increases as the plant matures, has a strong inhibitory relationship with cell wall digestibility [73,74] indicating the need to modify these components of the plant in order to enhance forage digestibility. Indeed, plant breeding to modify the plant cell wall and increase forage digestibility and reduce losses of nutrients to the environment are underway [75], but there may be tradeoffs with the fitness and persistence of modified plant species [76].

Maintenance energy requirements account for 70% of the feed used in a cow–calf production system, and feed and feed digestion account for large proportion of variable costs and greenhouse gas emissions, respectively. Thus, the net energy for maintenance was expected to have an influence on environmental and economic sustainability, and it did. Of the four strategies evaluated, decreasing NEm_Req had the greatest influence on DMI per kilogram weaned and return over variable costs through increasing the net energy for gain intake and growth of calves and reducing supplemental feed needs of cows while grazing. Thus, decreasing NEm_Req has the potential to enhance economic sustainability by increasing the output of weaned calves and decreasing purchased feed inputs. Additionally, decreasing NEm_Req has the potential to enhance environmental sustainability by increasing the output of weaned calves and decreasing the feed used by the breeding herd. However, NEm_Req had a moderate, negative influence on winter feed use that could result in less economically sustainable cow–calf production systems.

## 4. Conclusions

Improving multiple production parameters could enhance the environmental and economic sustainability of cow–calf production systems. Increasing harvested grazed forage per hectare could reduce feed expenses, while increasing forage digestibility and decreasing maintenance energy requirements could increase calf weaning weight to enhance economic sustainability. Increasing weaning weight through reduced maintenance energy requirements and increased forage digestibility had a strong influence on environmental sustainability; however, increased forage digestibility could negatively impact winter feed costs and economic sustainability. Thus, as long as optimum reproductive performance is achieved, a combination of improving maintenance energy requirements, forage yield, and forage digestibility has a strong ability to enhance environmental and economic sustainability with minor antagonisms.

A limitation of the current analysis is that although the economic benefit of the four strategies has been modeled, the estimated cost to make the improvements in the four strategies was not included in the model. The model does not account for the increased cost of plant breeding or purchasing improved forage varieties, expenses of animal breeding or purchasing bulls with a superior net energy for maintenance requirements, or infrastructure or management costs for improved grazing strategies. These were not included as they are difficult to estimate and would be even more difficult to vary with the magnitude of change in forage yield, forage digestibility, or the net energy for maintenance requirements with the distribution used in the model. Given the estimated impact on inputs and cow–calf performance from this analysis, future research should evaluate the feasibility of making improvements in the four strategies.

## Figures and Tables

**Table 1:** Descriptive statistics after 1000 iterations of parameters representing strategies to improve sustainable intensification of cow–calf production.

Variable	Mean	SD	Median	Min	Max
Herd base NEm requirement (NEm_Req)	0.0769	0.0019	0.0768	0.0709	0.0827
Forage TDN multiplicative factor (Forage_TDN)	1.000	0.021	1.000	0.937	1.088
Postpartum interval additive factor (PPI_Addend)	0.117	3.570	0.00	−12	11
Forage yield multiplicative factor (Forage_Yield)	1.000	0.035	0.999	0.911	1.096

SD = standard deviation; Min = minimum value; Max = maximum value.

**Table 2:** Descriptive statistics for production, efficiency, and economic variables from 1000 iterations of a 100-cow herd.

Variable	Mean	SD	Min	Max
Production				
Cycling in first 21 days, %	88.11	3.93	71.41	96.23
Postpartum interval, d	59.2	3.8	45.7	70.3
Pregnancy percentage, %	92.96	0.95	88.92	95.68
Calved in first 21 days, %	59.40	2.74	47.75	65.71
Median BCS at calving	5.8	0.3	5	6
Median BCS at weaning	4.4	0.4	4	6
Cull cow age, y	6.6	0.4	5.5	7.8
Replacement heifers required, %	15.74	0.89	13.16	19.06
Actual weaning weight of calves, kg	207.8	4.6	194.6	224.0
Supplement used, kg/cow	421.5	104.2	146.8	726.4
Winter feed used, kg/cow	1850.2	117.2	1486.9	2236.5
Total purchased feed, kg/cow	2271.7	61.7	2085.4	2454.6
Efficiency				
Grazing days per ha, d	10.5	0.4	9.3	11.8
Dry matter intake per kg weaned, kg/kg	26.49	0.70	24.46	28.78
Kg weaned per cow exposed, kg	172.4	4.6	158.1	189.0
Kg weaned per grazed ha, kg	60.8	1.5	56.1	67.2
Economic				
Revenue, USD/cow	708.12	12.36	675.94	754.60
Supplement cost, USD/cow	77.63	20.04	25.94	134.14
Winter feed cost, USD/cow	307.81	24.55	236.42	393.46
Purchased feed cost, USD/cow	385.44	22.56	320.99	470.54
Replacement cost, USD/cow	110.09	6.45	91.09	131.77
Interest expense, USD/cow	31.80	1.21	28.00	36.40
Total variable cost, USD/cow	667.82	25.23	592.83	762.38
Returns ^1^, USD/cow	40.30	29.00	−76.57	130.20
Returns ^1^, USD/grazed ha	14.28	10.25	−27.45	46.16
Annual ROI, %	8.01	4.58	−8.10	23.47

SD = standard deviation; Min = minimum value; Max = maximum value; BCS = body condition score, 1 = thin, 9 = obese. ^1^ Returns are calculated as revenue minus variable costs (i.e., returns over variable costs).

**Table 3:** Standardized regression coefficients between strategies (independent variables) and model outputs (dependent variables).

Output	NEm_Req ^1^	Forage_TDN	PPI	Forage_Yield
Production				
– Cycling in first 21 days	0.06	−0.02	−0.96	0.03
– Pregnancy percentage	0.03	−0.03	−0.59	0.05
– Calved in first 21 days	0.06	0.00	−0.77	0.03
– Cull cow age	0.04	−0.05	−0.51	0.08
– Replacement heifers required	−0.02	0.04	0.46	−0.03
– Actual weaning weight	−0.66	0.58	0.14	−0.07
– Supplement used per cow	0.71	−0.58	0.07	0.22
– Winter feed used per cow	−0.47	0.44	−0.12	−0.65
– Total purchased feed per cow	0.31	−0.15	−0.11	−0.86
Efficiency				
– Grazing days per hectare	0.11	−0.39	0.07	0.84
– Kg weaned per cow exposed	−0.53	0.47	−0.31	−0.04
– Kg weaned per grazed hectare	−0.54	0.47	−0.27	−0.05
– Dry matter intake per kg weaned	0.65	−0.50	0.20	0.13
Economic				
– Revenue per cow	−0.51	0.45	−0.14	−0.04
– Supplement cost per cow	0.70	−0.57	0.06	0.22
– Winter feed cost per cow	−0.35	0.32	−0.11	−0.47
– Purchased feed cost per cow	0.24	−0.15	−0.06	−0.32
– Replacement cost per cow	−0.22	0.20	0.37	−0.06
– Interest expense per cow	0.15	−0.09	0.04	−0.32
– Total variable cost per cow	0.15	−0.09	0.04	−0.31
– Returns per cow ^2^	−0.36	0.27	−0.09	0.26
– Returns per grazed hectare ^2^	−0.36	0.28	−0.10	0.26
– Annual ROI	−0.35	0.27	−0.11	0.26

^1^ NEm_Req = herd base net energy for maintenance requirement; Forage_TDN = forage TDN multiplicative factor; PPI = postpartum interval; Forage_Yield = forage yield multiplicative factor. ^2^ Returns are calculated as revenue minus variable costs (i.e., returns over variable costs).

**Table 4:** Standardized regression coefficients of variables on DMI per kilogram weaned, winter feed use per cow, and returns over variable costs per cow.

	Dependent Variable		
Independent Variables	DMI/kg Weaned	Winter Feed Use	Returns Over Variable Costs
Postpartum interval (PPI)	−0.003	−0.026	−0.073
Calved in first 21 days			−0.098
Cycling in first 21 days		−0.032	
Pregnancy percentage	−0.033	−0.013	
Replacement heifers required	0.184	−0.065	−0.154
Pounds cull cow sold			0.119
Calf mortality	0.173		
Grazing hectares per cow			−0.085
Grazing days per ha		−0.943	
Base NEm requirement (NEm_Req)	0.385	−0.290	
Total NEg intake	0.177		
Purchased feed per cow			−0.335
DMI per day per cow	0.028		
Forage TDN (Forage_TDN)	−0.238		
Forage yield per ha (Forage_Yield)	0.098	0.152	
Actual weaning weight	−0.609		
Kg weaned per cow exposed		0.135	0.444
Mean age of cull cows	−0.006		

## Data Availability

Data are available as a Microsoft Excel file in Supplementary Materials.

## Supplementary Materials

The following supporting information can be downloaded at: https://www.mdpi.com/article/10.3390/ani12030385/s1. Supplementary tables present model parameters [77,78,79,80,81,82,83,84,85,86,87,88,89,90,91,92,93,94,95,96,97,98,99,100,101,102,103,104,105,106]. Average model output variables for each of the 1000 iterations are available as a Microsoft Excel file: Data.xlsx.
