# Supplementary material for: Evaluation of Strategies to Improve the Environmental and Economic Sustainability of Cow–Calf Production Systems
Source: Animals (Basel). 2022 Feb 5;12(3):385. doi: 10.3390/ani12030385 (PMC8833546; doi:10.3390/ani12030385)
Supplement: Supplementary file 1 [file animals-12-00385-s001.zip › animals-1537843-supplementary.pdf]

Supplementary Material: Model Parameters [1]

Table S1. Initialization parameters.

Model Parameter	Distribution Type	Distribution Parameters	Reference	Notes
Iterations	Deterministic	1000	User-defined	The number of iterations the user wishes to run an *i* production year simulation
Initial breeding herd size at calving	Deterministic	100	User-defined	
Breeding herd size goal	Deterministic	100	User-defined	The breeding herd size the user wishes to achieve.
Years to herd size	Deterministic	1	User-defined	The number of years the user wishes to pass in achieving the breeding herd size goal.
Initial heifer replacement rate	Deterministic	0.125	User-defined (default from Wittum et al. [2], USDA [3], Cushman et al. [4], Ringwall [5], and expert opinion)	
Breeding season start	Deterministic	June 1st	User-defined	
Breeding season end	Deterministic	August 3	User-defined	

Reproduced with permission of the author [1]

Table S2. Four-year rolling average Angus genetic trend for birth weight and weaning weight from 1992 to 2018.

Year	Angus Birth Weight EBV (kg): Four Year Rolling Average	Angus Weaning Weight EBV (kg): Four Year Rolling Average
1992	1.66	10.9
1993	1.73	12.3
1994	1.75	13.6
1995	1.73	14.8
1996	1.70	16.1
1997	1.70	17.3
1998	1.70	18.6
1999	1.73	20.2
2000	1.73	21.6
2001	1.73	23.2
2002	1.73	24.5
2003	1.73	25.9
2004	1.70	27.2
2005	1.68	28.4
2006	1.66	29.7
2007	1.61	31.1
2008	1.59	32.7
2009	1.54	34.3
2010	1.50	35.9
2011	1.45	37.2
2012	1.41	38.6
2013	1.36	40.0
2014	1.32	41.3
2015	1.29	42.9
2016	1.25	44.5
2017	1.23	46.3
2018	1.18	48.1

Adapted from American Angus Association [6]
Reproduced with permission of the author [1]

Table S3. Genetic correlations.

Traits	Genetic Correlation	Reference
Weaning Weight: Mature Cow Weight	0.44	American Angus Association [6]
Birth Weight: Weaning Weight	0.29	American Angus Association [6]
Gestation: Birth Weight	0.30	Gregory et al. [7]
Milk Production: Mature Cow Weight	0.14	Morris and Wilton [8]

Reproduced with permission of the author [1]

Table S4. Manhattan, KS January through August Cumulative Precipitation.

Year	Jan- Aug Cumulative Precipitation, mm
1995	884.7
1996	599.7
1997	512.3
1998	613.4
1999	774.7
2000	391.9
2001	782.8
2002	468.1
2003	649.2
2004	826.5
2005	671.8
2006	665.5
2007	879.3
2008	840.0
2009	748.3
2010	678.2
2011	541.8
2012	443.5
2013	574.0
2014	606.5
2015	782.6
2016	787.6
2017	642.9
2018	507.5

From High Plains Regional Climate Center [9]

Reproduced with permission of the author [1]

Table S5. Diet total digestible nutrient (TDN) concentrations for winter feed, supplement and forage.

Diet	Month	TDN, %
Winter feed	Jan-Dec	57
Supplement	Jan-Dec	70
Bluestem Forage		
	Jan-Mar	43
	Apr	67
	May	63
	Jun	59
	Jul	55
	Aug	51
	Sep	47
	Oct-Dec	43

Calculated using estimates from National Academies of Science, Engineering, and Medicine [10] and Kuhl et al. [11].

Table S6 Daily maximum DMI as percent of SBW and animal production category.

Animal Category	Maximum Daily DMI (percent of SBW)
Post-weaning Non-pregnant Replacement Heifer	2.7
Bred Yearling Heifer	2.7
Two-Year-Old Cow	2.3
Three-Year-Old Cow	2.3
Mature Cow (>= 4-Years-Old)	2.2

SBW = shrunk body weight

Reproduced with permission of the author [1]

Table S7. BCS and corresponding body fat composition, percent of MSBW, and Mcal per kg of EBW loss and EBW gain.

BCS	Body Fat, % EBW	Percent of MSBW (BCS 5)	Mcal per kg EBW Loss	Mcal per kg EBW Gain
1	3.77	71.6	--	4.94
2	7.54	78.7	4.94	5.57
3	11.30	85.8	5.57	6.21
4	15.07	92.9	6.21	6.84
5	18.89	100.0	6.84	7.48
6	22.61	107.1	7.48	8.12
7	26.38	114.2	8.12	8.74
8	30.15	121.3	8.74	9.36
9	33.91	128.4	9.36	--

Adapted from National Academies of Science, Engineering, and Medicine [10]

MSBW = mature shrunk body weight; EBW = empty body weight

Table S8. Maximum winter feed intake by animal production category.

Animal Production Category	Maximum Winter Feed Intake, kg/d
Post-weaning Non-pregnant Replacement Heifer	13.0
Bred Yearling Heifer	13.0
Two-Year-Old Cow	16.0
Three-Year-Old Cow	16.0
Mature Cow (>= 4-Years-Old)	16.0

Reproduced with permission of the author [1]

Table S9. Reproductive cyclicity.

Model Parameter	Distribution Type	Distribution Parameters	Reference
Postpartum Interval Primiparous Cows, d			Ciccioli et al. [12], Berardinelli et al. [13], Endecott et al. [14], and expert opinion
BCS 1	Pert	(350, 350, 350)	
BCS 2	Pert	(135, 150, 165)	
BCS 3	Pert	(85, 100, 115)	
BCS 4	Pert	(65, 80, 95)	
BCS 5	Pert	(55, 70, 85)	
BCS 6	Pert	(45, 60, 75)	
BCS 7	Pert	(30, 45, 60)	
BCS 8	Pert	(30, 45, 60)	
BCS 9	Pert	(30, 45, 60)	

BCS = body condition score (1 = very thin, 9 = obese)

Reproduced with permission of the author [1]

Table S9 (*cont*). Reproductive cyclicity.

Model Parameter	Distribution Type	Distribution Parameters	Reference
PostPartum Interval Multiparous Cows, d			Graham [15],Rutter and Randel [16], Houghton et al. [17], Cushman et al. [18], Lents et al. [19], and expert opinion
BCS 1	Pert	(350, 350, 350)	
BCS 2	Pert	(135, 150, 165)	
BCS 3	Pert	(75, 90, 105)	
BCS 4	Pert	(55, 70, 85)	
BCS 5	Pert	(45, 60, 75)	
BCS 6	Pert	(35, 50, 65)	
BCS 7	Pert	(30, 35, 50)	
BCS 8	Pert	(30, 35, 50)	
BCS 9	Pert	(30, 35, 50)	

Dystocia Probability per Parturition			McDermott et al. [20], USDA [21], and expert opinion
Multiparous Cow	Normal	(0.05, 0.01, lower = 0)	
Primiparous Cow-Calf birthweight < 40.82 kg	Normal	(0.08, 0.01, lower = 0)	
Primiparous Cow-Calf birthweight >= 40.82 kg	Normal	(0.5, 0.01, lower = 0)	

BCS = body condition score (1 = very thin, 9 = obese)

Reproduced with permission of the author [1]

Table S9 (*cont*). Reproductive cyclicity.

**Model Parameter**	**Distribution Type**	**Distribution Parameters**	**Reference**
Additional PPI resulting from dystocia, d	Normal	(10, 2, lower = 0)	Doornbos et al. [22], Bellows et al. [23], and expert opinion.
Pregnancy probability at d equal to estrous cycle length after breeding			Spell et al. [24], Chagas et al. [25], Aherin et al. [26], and expert opinion
Heifers	Normal	(0.71, 0.01, upper = 0.8)	Cundiff et al. [27]
Primiparous Cows	Normal	(0.61, 0.01, upper = 0.8)	Cundiff et al. [27]
Multiparous Cows	Normal	(0.71, 0.01, upper = 0.8)	Cundiff et al. [27]

Reproduced with permission of the author [1]

Table S9 (*cont*). Reproductive cyclicity.

Model Parameter	Distribution Type	Distribution Parameters	Reference
Daily mean probability of returning to cyclicity after establishing pregnancy			
d 25 to d 45	Normal	(0.002, 0.0002, lower = 0)	Whittier et al. [28], Lamb et al. [29], Aherin et al. [26], and expert opinion.
d 46 to d 65	Normal	(0.0005, 0.00002, lower = 0)	Whittier et al. [28], Lamb et al. [29], Aherin et al. [26], and expert opinion.
d > 65	Normal	(0.0001, 0.00002, lower = 0)	Dziuk and Bellows [30], van Wagtendonk-de Leeuw et al. [31], Aherin et al. [26], and expert opinion
Individual Gestation length	Normal	(285, 7)	Expert opinion

Reproduced with permission of the author [1]

Table S10. Culling.

Model Parameter	Distribution Type	Distribution Parameters	Reference
Pregnancy determination (days after breeding season end)	Deterministic	60	User-defined
Age of oldest calf at weaning, d	Deterministic	220	User-defined
Maximum cow age	Deterministic	13	User-defined
Minimum culling percentage by cow age (involuntary and voluntary combined), yr		culls within age/exposed within age, percent	Wittum et al. [2], USDA [3], Cushman et al. [4], Ringwall [5], and expert opinion
1	Deterministic	5	
2	Deterministic	10	
3	Deterministic	6	
4	Deterministic	6	
5	Deterministic	6	
6	Deterministic	6	
7	Deterministic	6	
8	Deterministic	6	
9	Deterministic	8	
10	Deterministic	10	
11	Deterministic	40	
12	Deterministic	50	
13	Deterministic	100	

Reproduced with permission of the author [1]

Table S11. Morbidity and mortality.

Model Parameter	Distribution Type	Distribution Parameters	Reference
Daily probability of preweaned calf morbidity			Wittum et al. [2], Sanderson and Dargatz [32], USDA [3], and expert opinion
Dystocia and neonatal period (d 1-3 after parturition)	Normal	(0.01, 0.005, lower = 0)	
No dystocia and neonatal period (d 1-3 after parturition)	Normal	(0.005, 0.001, lower = 0)	
Dystocia and post-neonatal period to weaning	Normal	(0.0004, 0.00001, lower = 0)	
No dystocia and post-neonatal period to weaning	Normal	(0.0002, 0.00001, lower = 0)	

Reproduced with permission of the author [1]

Table S11 (*cont*). Morbidity and mortality.

Model Parameter	Distribution Type	Distribution Parameters	Reference
Daily probability of preweaned calf mortality			Laster and Gregory [33], Patterson et al. [34], Wittum et al. [2], USDA [3], and expert opinion
Dystocia, no morbidity, and neonatal period	Normal	(0.06, 0.005, lower = 0)	
Dystocia, morbidity, and neonatal period	Normal	(0.1, 0.0005, lower = 0)	
No dystocia, no morbidity, and neonatal period	Normal	(0.01, 0.001, lower = 0)	
No dystocia, morbidity, and neonatal period	Normal	(0.05, 0.001, lower = 0)	
Dystocia, no morbidity, and post-neonatal period to weaning	Normal	(0.0001, 0.00001, lower = 0)	
Dystocia, morbidity, and post-neonatal period to weaning	Normal	(0.001, 0.0001, lower = 0)	
No dystocia, no morbidity, and post-neonatal period to weaning	Normal	(0.0001, 0.00001, lower = 0)	
No dystocia, morbidity, and post-neonatal period to weaning	Normal	(0.0005, 0.0001, lower = 0)	

Reproduced with permission of the author [1]

Table S11 (*cont*). Morbidity and mortality.

Model Parameter	Distribution Type	Distribution Parameters	Reference
Daily probability of postweaning mortality			USDA [3], and expert opinion
Dystocia at birth	Normal	(0.00005, 0.00001,lower = 0)	
No Dystocia at birth	Normal	(0.000025, 0.00001,lower = 0)	
Daily probability of mature mortality	Normal	(0.000025, 0.00001,lower = 0)	USDA [3], and expert opinion
Percent reduction in WW from morbidity	Normal	(0.065, 0.0065)	Wittum et al. [2]

WW = weaning weight

Reproduced with permission of the author [1]

Table S12. Calf growth**.**

Model Parameter	Distribution Type	Distribution Parameters	Reference
Calf birthweights			
Bull calf, two-year-old dam mean birthweight adjustment, kg	Deterministic	-3.63	Beef Improvement Federation [35]
Bull calf, three-year-old dam mean birthweight adjustment, kg	Deterministic	-2.27	Beef Improvement Federation [35]
Bull calf, four-year-old dam mean birthweight adjustment, kg	Deterministic	-0.91	Beef Improvement Federation [35]
Bull calf, eleven-year-old and older dam mean birthweight adjustment, kg	Deterministic	-1.36	Beef Improvement Federation [35]

Reproduced with permission of the author [1]

Table S12 (*cont*). Calf growth.

Model Parameter	Distribution Type	Distribution Parameters	Reference
Calf birthweights			
Heifer calf, two-year-old dam mean birthweight adjustment, kg	Deterministic	-3.17	Beef Improvement Federation [35]
Heifer calf, three-year-old dam mean birthweight adjustment, kg	Deterministic	-2.27	Beef Improvement Federation [35]
Heifer calf, four-year-old dam mean birthweight adjustment, kg	Deterministic	-0.91	Beef Improvement Federation [35]
Heifer calf, eleven-year-old and older dam mean birthweight adjustment, kg	Deterministic	-1.36	Beef Improvement Federation [35]

Reproduced with permission of the author [1]

Literature Cited

1. Aherin, D.G. Stochastic Systems Model Assessment of Historical Cow-Calf Biological and Economic Efficiency for Different Mature Cow Weight and Peak Lactation Combinations in the Kansas Flint Hills. Ph.D. Thesis, Kansas State University, Manhattan, KS, USA, 2020.
2. Wittum, T.E.; Salman, M.D.; King, M.E.; Mortimer, R.G.; Odde, K.G.; Morris, D.L. The Influence of Neonatal Health on Weaning Weight of Colarado, USA Beef Calves. *Prev. Vet. Med.* **1994**, *19*, 15–25, doi:10.1016/0167-5877(94)90011-6.
3. USDA *Part IV: Reference of Beef Cow-Calf Management Practices in the United States, 2007-08*; Beef 2007-08; USDA:APHIS:VS:CEAH: Fort Collins, CO, 2010;
4. Cushman, R.A.; Kill, L.K.; Funston, R.N.; Mousel, E.M.; Perry, G.A. Heifer Calving Date Positively Influences Calf Weaning Weights through Six Parturitions. *J. Anim. Sci.* **2013**, *91*, 4486–4491, doi:10.2527/jas.2013-6465.
5. Ringwall, K. BeefTalk: Age and Weight Are Cow Herd Dynamics Available online: http://www.drovers.com/news/beeftalk-age-and-weight-are-cow-herd-dynamics (accessed on 8 December 2021).
6. American Angus Association Heritabilities Available online: https://www.angus.org/Nce/Heritabilities (accessed on 10 December 2019).
7. Gregory, K.E.; Cundiff, L.V.; Koch, R.M. Genetic and Phenotypic (Co)Variances for Production Traits of Intact Male Populations of Purebred and Composite Beef Cattle. *J. Anim. Sci.* **1995**, *73*, 2227–2234, doi:10.2527/1995.7382227x.
8. Morris, C.A.; Wilton, J.W. Influence of Body Size on the Biological Efficiency of Cows: A Review. *Can. J. Anim. Sci.* **1976**, *56*, 613–647, doi:10.4141/cjas76-076.
9. High Plains Regional Climate Center Manhattan Agronomy Farm Available online: https://hprcc.unl.edu/index.php (accessed on 15 November 2019).
10. National Academies of Sciences, Engineering, and Medicine *Nutrient Requirements of Beef Cattle: Eighth Revised Edition*; Animal Nutrition Series; The National Academies Press: Washington, DC, USA, 2016; ISBN 978-0-309-31702-3.
11. Kuhl, G.L.; Simms, D.D.; Bandyk, C.A. *Nutritional Composition of Feedstuffs for Beef Cattle*; Kansas State University Agricultural Experiment Station and Cooperative Extension Service: Manhattan, KS, USA, 1993; p. 4;.
12. Ciccioli, N.H.; Wettemann, R.P.; Spicer, L.J.; Lents, C.A.; White, F.J.; Keisler, D.H. Influence of Body Condition at Calving and Postpartum Nutrition on Endocrine Function and Reproductive Performance of Primiparous Beef Cows1,2. *J. Anim. Sci.* **2003**, *81*, 3107–3120, doi:10.2527/2003.81123107x.
13. Berardinelli, J.G.; Joshi, P.S.; Tauck, S.A. Postpartum Resumption of Ovarian Cycling Activity in First-Calf Suckled Beef Cows Exposed to Familiar or Unfamiliar Bulls. *Anim. Reprod. Sci.* **2005**, *90*, 201–209, doi:10.1016/j.anireprosci.2005.02.005.
14. Endecott, R.; Cox, S.; Petersen, M.K. Impacts of Supplemental Glucogenic Precursors and Cow Age on Postpartum Range Cow Performance. *Proc. West. Sect. Am. Soc. Anim. Sci.* **2007**, *58*, 352–357.
15. Graham, J. The Effect of Body Condition of Beef Cows at Calving and Post Calving Nutrition on Calf Growth Rate and Cow Fertility. *Proc. Aust. Soc. Anim. Prod.* **1982**, *14*, 309–312.
16. Rutter, L.M.; Randel, R.D. Postpartum Nutrient Intake and Body Condition: Effect on Pituitary Function and Onset of Estrus in Beef Cattle. *J. Anim. Sci.* **1984**, *58*, 265–274, doi:10.2527/jas1984.582265x.
17. Houghton, P.L.; Lemenager, R.P.; Horstman, L.A.; Hendrix, K.S.; Moss, G.E. Effects of Body Composition, Pre- and Postpartum Energy Level and Early Weaning on Reproductive Performance of Beef Cows and Preweaning Calf Gain. *J. Anim. Sci.* **1990**, *68*, 1438–1446, doi:10.2527/1990.6851438x.
18. Cushman, R.A.; Allan, M.F.; Thallman, R.M.; Cundiff, L.V. Characterization of Biological Types of Cattle (Cycle VII): Influence of Postpartum Interval and Estrous Cycle Length on Fertility12. *J. Anim. Sci.* **2007**, *85*, 2156–2162, doi:10.2527/jas.2007-0136.
19. Lents, C.A.; White, F.J.; Ciccioli, N.H.; Wettemann, R.P.; Spicer, L.J.; Lalman, D.L. Effects of Body Condition Score at Parturition and Postpartum Protein Supplementation on Estrous Behavior and Size of the Dominant Follicle in Beef Cows,. *J. Anim. Sci.* **2008**, *86*, 2549–2556, doi:10.2527/jas.2008-1114.
20. McDermott, J.J.; Allen, O.B.; Martin, S.W.; Alves, D.M. Patterns of Stillbirth and Dystocia in Ontario Cow-Calf Herds. *Can. J. Vet. Res.* **1992**, *56*, 47–55.

21. USDA *Part III: Changes in the U.S. Beef Cow-Calf Industry, 1993-2008*; Beef 2007-08; USDA:APHIS:VS:CEAH: Fort Collins, CO, USA, 2009;
22. Doornbos, D.E.; Bellows, R.A.; Burfening, P.J.; Knapp, B.W. Effects of Dam Age, Prepartum Nutrition and Duration of Labor on Productivity and Postpartum Reproduction in Beef Females. *J. Anim. Sci.* **1984**, *59*, 1–10, doi:10.2527/jas1984.5911.
23. Bellows, R.A.; Short, R.E.; Staigmiller, R.B.; Milmine, W.L. Effects of Induced Parturition and Early Obstetrical Assistance in Beef Cattle. *J. Anim. Sci.* **1988**, *66*, 1073–1080, doi:10.2527/jas1988.6651073x.
24. Spell, A.R.; Beal, W.E.; Corah, L.R.; Lamb, G.C. Evaluating Recipient and Embryo Factors That Affect Pregnancy Rates of Embryo Transfer in Beef Cattle. *Theriogenology* **2001**, *56*, 287–297, doi:10.1016/S0093-691X(01)00563-5.
25. Chagas e Silva, J.; Lopes da Costa, L.; Robalo Silva, J. Plasma Progesterone Profiles and Factors Affecting Embryo-Fetal Mortality Following Embryo Transfer in Dairy Cattle. *Theriogenology* **2002**, *58*, 51–59, doi:10.1016/S0093-691X(02)00906-8.
26. Aherin, D.G.; Bormann, J.M.; Heier Stamm, J.L.; MacNeil, M.D.; Weaber, R.L. Decision-Making Tools: Stochastic Simulation Model Accounting for the Impacts of Biological Variation on Success of Bovine Embryo Transfer Programs1. *Transl. Anim. Sci.* **2018**, *2*, 451–462, doi:10.1093/tas/txy087.
27. Cundiff, L.V.; Gregory, K.E.; Koch, R.M. Effects of Heterosis on Reproduction in Hereford, Angus and Shorthorn Cattle2. *J. Anim. Sci.* **1974**, *38*, 711–727, doi:10.2527/jas1974.384711x.
28. Whittier, J.C.; Caldwell, R.W.; Anthony, R.V.; Smith, M.F.; Morrow, R.E. Effect of a Prostaglandin F2α Injection 96 Hours after Introduction of Intact Bulls on Estrus and Calving Distribution of Beef Cows. *J. Anim. Sci.* **1991**, *69*, 4670–4677, doi:10.2527/1991.69124670x.
29. Lamb, G.C.; Dahlen, C.R.; Vonnahme, K.A.; Hansen, G.R.; Arseneau, J.D.; Perry, G.A.; Walker, R.S.; Clement, J.; Arthington, J.D. Influence of a CIDR Prior to Bull Breeding on Pregnancy Rates and Subsequent Calving Distribution. *Anim. Reprod. Sci.* **2008**, *108*, 269–278, doi:10.1016/j.anireprosci.2007.08.012.
30. Dziuk, P.J.; Bellows, R.A. Management of Reproduction of Beef Cattle, Sheep and Pigs. *J. Anim. Sci.* **1983**, *57*, 355–379, doi:10.2527/animalsci1983.57Supplement_2355x.
31. van Wagtendonk-de Leeuw, A.M.; Mullaart, E.; de Roos, A.P.W.; Merton, J.S.; den Daas, J.H.G.; Kemp, B.; de Ruigh, L. Effects of Different Reproduction Techniques: AI, Moet or IVP, on Health and Welfare of Bovine Offspring. *Theriogenology* **2000**, *53*, 575–597, doi:10.1016/S0093-691X(99)00259-9.
32. Sanderson, M.W.; Dargatz, D.A. Risk Factors for High Herd Level Calf Morbidity Risk from Birth to Weaning in Beef Herds in the USA. *Prev. Vet. Med.* **2000**, *44*, 97–106, doi:10.1016/S0167-5877(99)00112-9.
33. Laster, D.B.; Gregory, K.E. Factors Influencing Peri- and Early Postnatal Calf Mortality. *J. Anim. Sci.* **1973**, *37*, 1092–1097, doi:10.2527/jas1973.3751092x.
34. Patterson, D.J.; Bellows, R.A.; Burfening, P.J.; Carr, J.B. Occurrence of Neonatal and Postnatal Mortality in Range Beef Cattle. I. Calf Loss Incidence from Birth to Weaning, Backward and Breech Presentations and Effects of Calf Loss on Subsequent Pregnancy Rate of Dams. *Theriogenology* **1987**, *28*, 557–571, doi:10.1016/0093-691X(87)90273-1.
35. Beef Improvement Federation *Guidelines for Uniform Beef Improvement Programs*; Beef Improvement Federation: North Mississippi Research and Extension Center, Verona, MS, USA, 2010;
